# Adverse Childhood Experiences and Intergenerational Support to Aging Parents

**DOI:** 10.1093/geroni/igaf122.2107

**Published:** 2025-12-31

**Authors:** Jooyoung Kong, Amy Liu, Stephanie Robert, Xing Zing

**Affiliations:** University of Wisconsin–Madison, Madison, Wisconsin, United States; University of Wisconsin–Madison, Madison, Wisconsin, United States; University of Wisconsin–Madison, Madison, Wisconsin, United States; Arizona State University, Phoenix, Arizona, United States

## Abstract

**Background and Objective:**

Using the support bank model, the current study examined aging mothers’ receipt of support from their adult children, focusing on the roles of adult children’s emotional closeness and aging mothers’ needs. We also explored the long-term impacts of adult children’s reports of adverse childhood experiences (ACEs) on intergenerational support patterns.

**Methods:**

Data were drawn from 2,162 aging mothers (average age 63 years) who participated in the Add Health Parent Study Phase I and their adult-child respondents from Waves I, III and IV of the National Longitudinal Study of Adolescent to Adult Health. We conducted a series of logistic regressions to predict a binary outcome of adult children’s assistance (financial and instrumental) to aging mothers.

**Results:**

Mothers’ poor health and not being married or partnered were significantly associated with receiving financial and instrumental assistance from their adult children. Among adult daughters, the number of ACEs interacted with mothers’ depressive symptoms, such that having a greater number of ACEs exacerbated the negative impact of mothers’ depressive symptoms on the receipt of assistance. Specifically, with each additional adult child’s ACE, the effect of mothers’ depressive symptoms on the odds of receiving assistance weakened – decreasing from 60% to 44%.

**Conclusions:**

Intergenerational support for aging mothers may be hampered by adult daughters’ ACEs histories. Our focus on the life course factor of ACEs can contribute to a holistic view of later-life intergenerational support patterns. Future research could further explore the changes or stability in family dynamics for individuals with ACEs histories.

